# The Effect of Controlled-Release Carvacrol on Safety and Quality of Blueberries Stored in Perforated Packaging

**DOI:** 10.3390/foods10071487

**Published:** 2021-06-26

**Authors:** Xiuxiu Sun, Randall G. Cameron, Anne Plotto, Tian Zhong, Christopher M. Ference, Jinhe Bai

**Affiliations:** 1 Pacific Basin Agricultural Research Center, United States Department of Agriculture, Agricultural Research Service, 64 Nowelo St, Hilo, HI 96720, USA; 2 Horticultural Research Laboratory, United States Department of Agriculture, Agricultural Research Service, 2001 S. Rock Rd, Ft. Pierce, FL 34945, USA; randall.cameron@usda.gov (R.G.C.); anne.plotto@usda.gov (A.P.); Christopher.Ference@usda.gov (C.M.F.); 3 Faculty of Medicine, Macau University of Science and Technology, Avenida Wai Long, Taipa, Macau 999078, China; tzhong@must.edu.mo

**Keywords:** microcapsules, antimicrobial activity, sensory evaluation, volatile compound

## Abstract

The objective of this research was to evaluate the use of a controlled-release carvacrol powder to delay storage decay and maintain the safety of blueberries. The controlled-release carvacrol powder was a microcapsule of carvacrol (11% (*w*/*w*) active carvacrol) surrounded by a pectin/sodium alginate matrix. The microcapsules were packed in an air-permeable pouch, and then attached to the top of a clamshell filled with blueberries. The blueberries, inoculated with *Escherichia coli* or *Colletotrichum acutatum*, or non-inoculated control, were monitored for microbial growth and quality for 10 days at 10 °C and 5 days at 20 °C. Three treatments were compared: controlled-release microencapsulated carvacrol, non-encapsulated carvacrol, and control. The results showed that both the microencapsulated carvacrol and the non-encapsulated carvacrol treatments significantly reduced the populations of yeast and mold, and of *E. coli* and mesophilic aerobic bacteria. The microencapsulated carvacrol treated berries retained better quality due to significantly lower weight loss than control after 10 days at 10 °C. Sensory panelists found that the microencapsulated carvacrol berries had significantly higher overall blueberry flavor and lower discernible off-flavor in comparison with the non-encapsulated treatment after 3 days at 20 °C. The fruit internal quality, including total soluble solids content (SSC), and titratable acidity (TA), was not significantly affected by any treatment. These results indicate that pectin/sodium alginate controlled-release microencapsulated carvacrol can be used for the preservation of blueberries or other small fruit.

## 1. Introduction

Blueberries have been proven to be a rich source of phenolic compounds, dietary fiber, and vitamin C along with processing strong antioxidant activities [1]. However, blueberries are highly perishable due to the rapid physical damage caused by precipitous water loss and common microbial decay [2]. To maintain the safety and quality of blueberries, one proven successful postharvest technique is the application of edible films and coatings containing certain essential oils that naturally have strong antimicrobial activity [2,3,4].

Essential oils are naturally occurring chemicals extracted from plant seeds, leaves, and fruits [3]. Carvacrol is an antimicrobial compound which is a constituent of the essential oil of *Origanum vulgare* (oregano), as well as a major constituent of oil of *Thymus vulgaris* (thyme), and is also obtainable from *Citrus aurantium bergamia* (wild bergamot), *Lepidium flavum* (pepperwort), and *Lippia berlandieri Schauer* (Mexican Oregano) [5,6]. The in vitro antimicrobial activities of carvacrol are recognized to be effective against a variety of foodborne pathogenic and spoilage microorganisms [7]. Carvacrol exhibited strong antimicrobial activity against *Escherichia coli* O157:H7 in broth and rumen systems [8], and *Listeria monocytogenes* in steak tartare [9]. The antifungal activity of carvacrol against *Botrytis cinerea* [10], *Penicillium digitatum* [2], and *Mucor piriformis* [11] has also been investigated.

Even though carvacrol shows strong antimicrobial and antifungal activities, its strong odor and hydrophobic properties limit its direct application to foods [5]. One technique to mitigate the limitations of carvacrol is microencapsulation [12]. Microencapsulation is a delivery system for sensitive bioactive compounds (e.g., essential oils), which is usually employed to support the stability of the bioactive agents, to maximize their retention and to control the release of the encapsulated bioactive compounds at the target locations [13,14].

In this research, microencapsulated carvacrol was tested on stored blueberries. The major objective of this research was to investigate the effect of encapsulated carvacrol on blueberries inoculated with the food pathogen *E. coli* and postharvest pathogen *C. accutatum* and observe any secondary effects on fruit quality. Firmness, weight loss, soluble solids, titratability, as well as on the consumer-related sensory properties of blueberries, were measured. 

## 2. Materials and Methods

### 2.1. Materials

Blueberries (*Vaccinium corymbosum*, cultivar ‘Bluecrop’) were purchased from a local retailer, brought to the U.S. Horticultural Research Laboratory (Fort Pierce, FL, USA), and used immediately after purchase or stored at 5 °C overnight. Citrus pectin (≥74% degree of methylation), sodium alginate, carvacrol (99%, natural, and generally recognized as safe (GRAS)), and Tween-80 were obtained from Sigma Aldrich (St. Louis, MO, USA).

### 2.2. Microcapsule Powder Preparation

Equal weights of pectin and alginate polymers were mixed with distilled water and gently shaken in a water bath at room temperature (about 20 °C) overnight to create a 3% (*w*/*v*) pectin-alginate solution. A mixture of 5 mL Tween^®^ 80 and 1 mL carvacrol was introduced by slowly dripping into 100 mL of the blended biopolymer solution. Emulsification was achieved with a homogenizer (model Bio-Den Series Pro200, PRO Scientific, Oxford, CT, USA) at 12,000 rpm for 5 min. The emulsion was then spray-dried using a Mini Spray Dryer (model B-290, BUCHI Corporation, New Castle, DE, USA) with a two-fluid nozzle consisting of a 1.5 mm diameter nozzle cap with a 0.7 mm diameter nozzle tip hole. The operation parameters were set according to our previous research [14]. Briefly, the feed rate, inlet air temperature, aspirator, and air flow rate were 7 mL min^−1^, 100 °C, 100%, and 26 L min^−1^, respectively. 

### 2.3. Inoculum Preparation

Inoculum was made from strains of *E. coli* K12 MDD333 and *C. acutatum* Corda kept at −80 °C on *E. coli* agar (ECA) (EC Broth with 2% agar), and potato dextrose agar (PDA) plugs in cryoprotectant (10% glycerol), respectively. Frozen plugs of *E. coli* were grown on ECA for 24 h at 37 °C and subsequently sub-cultured for an additional 24 h at the same temperature. Cultures were identified by streaking plates of Levine eosin methylene blue (EMB) indicator agar with the bacteria, and incubating the plates at 37 °C for 24 h. Cultures which were reflective metallic green on EMB agar were confirmed to be *E. coli*. The PDA plugs were re-cultured on PDA at 25 °C for 10 d and identified by spore production. The *E. coli* cells and *C. acutatum* spores were scraped from the ECA and PDA plates, respectively, and suspended in 1 L of 20 °C sterile deionized (DI) water. The final concentrations of *E. coli* and *C. acutatum* populations in the inoculation broth were each 5 log CFU mL^−1^. 

### 2.4. Inoculation, Treatment, and Storage

One liter of inoculum, including both *E. coli* and *C. acutatum*, was administered via a spray bottle onto 5 kg of fresh blueberries in a 10-L autoclavable metal container. After 3 min, the fruit were removed from the container and air-dried on sterilized steel mesh for 2 h before the blueberries (200 g) were placed in one-pound clamshells (OSU #1, Packaging Plus, Yakima, WA, USA). These clamshells have a 0.44% opening ratio in comparison with 2.83% in the typical commercial clamshells [15]. The microencapsulated carvacrol pouch was made with 0.5 g of powder (11% (*w*/*w*) active carvacrol) wrapped in Miracloth (Millipore, Billerica, MA, USA), which allowed the carvacrol vapor to release and diffuse gradually and continuously. Pouches were affixed to the underside of clamshell lids with two-sided adhesive tape. For the carvacrol direct application, 55 mg of carvacrol (equivalent to 11% active carvacrol) were pipetted on to a 2 cm^2^ piece of filter paper, which was then attached in the same manner as the pouch. Blueberries in the clamshells without carvacrol were used as a control. The blueberries were stored at 10 °C for 10 days, and 20 °C for 5 days. The microbial populations and fruit weight loss were evaluated at day 0, 4, 7, and 10 at 10 °C, and day 1, 3, and 5 at 20 °C. The firmness and sensory properties were measured at day 7 at 10 °C and day 3 at 20 °C on a different set of non-inoculated fruit subjected to the same treatments. The fruit internal quality, including total soluble solids content (SSC) and titratable acidity values (TA), was also determined using previous methods [16]. Each treatment included three replicates.

### 2.5. Microbiological Analyses

The experiments were conducted in a biosafety level 2 sterile laboratory setting. Ten samples from each clamshell-replicate were disturbed in sterile phosphate buffer (99 mL; 0.01 M titrated to pH 7.2) for 1 h in an autoclavable 13 oz sampling container (WhirlPak, Madison, WI, USA) on an orbital platform shaker (Innova 2100, New Brunswick Scientific, New Brunswick, NJ, USA). Serial dilutions of buffered fruit rinse were spiral-plated on PDA for yeast and mold count (YMC) and on plate count agar (PCA; Difco BD, Fisher Scientific, Hampton, NH, USA) for enumeration of total bacteria count (TBC) using an Eddy Jet model automatic spiral plater with disposable sterile tips (Neutec Group Inc., Farmingdale, NY, USA).The incubation of PCA plates was for 2 days at 25 °C while PDA plates were for 3 days, and all subsequent microbial growth was read using a ProtoCOL model optical microbial colony counting machine (Synoptics, Ltd., Cambridge, UK). All tests were performed in triplicate.

### 2.6. Firmness and Weight Loss

Blueberry firmness was measured by a Texture Analyzer (model TA. XT2i, Stable Micro Systems, Inc., Surrey, UK) and expressed as Newtons (N). Every berry fruit was compacted between two parallel plates. The pre-speed, test speed, post-speed, and distance were 5 mm s^−1^, 0.5 mm s^−1^, 10 mm s^−1^, and 2.5 mm s^−1^, respectively. The trigger type was auto with a trigger force of 0.049 N. In total, 20 non-inoculated blueberries were used from each clamshell replicate and three clamshells were used per treatment.

The weight of each clamshell of fruit was measured on day 0, and on the sampling days. Values were expressed as percentage of weight loss per initial fruit weight.

### 2.7. Sensory Evaluation

Four to six blueberries were placed on plates identified with 3-digit codes. Each number coded plate was served at room temperature for sensory evaluation. Ten panelists—laboratory members trained to evaluate the consumer-related sensory properties of fresh fruit—were asked to rate samples for intensity of blueberry flavor and strength of off-flavor using a 0 to 10 scale (0-none; 10-strong) [17]. To make up for fruit-to-fruit variability, panelists were trained to taste two pieces of fruit at a time and evaluated no fewer than four fruit in total from each number-coded plate.

### 2.8. Carvacrol and Overall Volatile Profile Measured by Using GC-MS

A headspace solid-phase microextraction gas chromatography-mass spectrometry (HS-SPME-GC-MS) system was used to determine carvacrol and overall volatile profile in the fruit samples following the previous method [16]. Briefly, 4 blueberries (about 8 g), cut in to 4 pieces of each, were sealed in a 20-mL glass vial; a 2-cm SPME fiber (50/30 μm DVB/Carboxen/PDMS; Supelco, Bellefonte, PA) was inserted in the vial to extract the headspace volatiles for 60 min at 40 °C. Volatiles were separated by a DB-5 column (60 m × 0.25 mm i.d., 1.00 μm film thickness; J&W Scientific, Folsom, CA, USA) equipped GC, and identified by a MS detector (GC-MS, Model 6890, Agilent, Santa Clara, CA, USA). The concentration of residual carvacrol in blueberries was calculated using a standard curve with four levels (2 ng L^−1^ to 2 mg L^−1^) of standard carvacrol dissolved in acetone.

### 2.9. Statistical Analysis

Data were analyzed using analysis of variance (ANOVA) with JMP (version 11.2.0; SAS Institute, Cary, NC, USA). Mean separation was determined by Student’s *t* test. Significance was defined at *p* < 0.05. Each experimental treatment contained three replicated clamshells.

## 3. Results and Discussion

### 3.1. Microbial Populations

**Figure 1:**
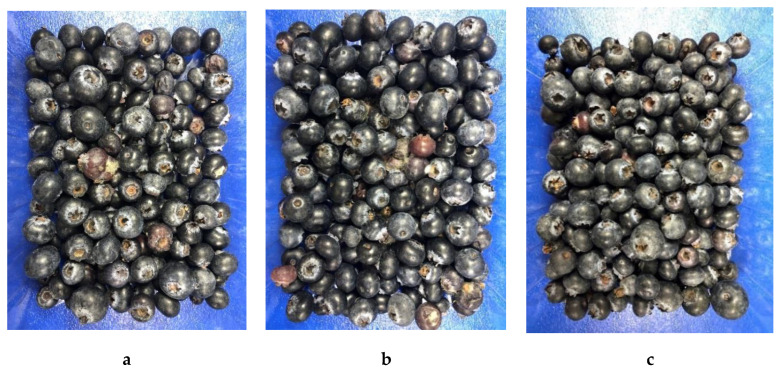
Effect of pectin-sodium alginate microencapsulated carvacrol (**c**) and non-encapsulated carvacrol (**b**) in comparison with control (**a**) on appearance of inoculated blueberries with *C. acutatum* after 10 days storage at 10 °C.

Blueberries, inoculated with *E. coli* or *C. acutatum*, or non-inoculated, were monitored for surviving microbial populations and overall fruit quality for up to 10 days at 10 °C and for up to 5 days at 20 °C. The results showed that both the encapsulated and non-encapsulated carvacrol treatments reduced the populations of yeasts, molds, *E. coli*, and total mesophilic aerobic bacteria (Table 1). No active *E. coli* were detected surviving on either encapsulated or non-encapsulated carvacrol treated berries at 7 days of storage at 10 °C and 3 days of storage at 20 °C (Table 1). Populations of yeast, mold, and mesophilic aerobic bacteria recovered from encapsulated and non-encapsulated carvacrol treated berries were significantly lower than control after 10 days storage at 10 °C and 5 days storage at 20 °C (Table 1). Encapsulated carvacrol treated berries had less visible mold than non-encapsulated carvacrol treated and control fruit after 10 days storage at 10 °C (Figure 1).

Several explanations for the antimicrobial activity of carvacrol are possible. The antimicrobial mode of action for carvacrol may be associated to its hydrophobicity and consequent ability to move across the fungal cell membrane, which additionally affects cell pH homeostasis and equilibrium of inorganic ions, further disturbing intracellular structures [18,19]. The antimicrobial activity of carvacrol may be generated through a mechanism of action where formaldehyde and its reaction products, such as formaldemethone, are produced by the microorganism [20,21]. Carvacrol may be inhibiting the activity of cell wall enzymes of microorganisms [22].

In previous research, carvacrol demonstrated antibacterial activity against *E. coli* [19,23] and nonselective antifungal activity against three *Colletotrichum* species [20]. Carvacrol encapsulated in micellar nonionic surfactant solutions extensively inhibited the growth of *E. coli* [24]. A chitosan-carvacrol coating substantially decreased the population of *E. coli* and yeast and mold on blueberries [2]. Gum arabic microcapsules containing carvacrol inhibited the growth of *E. coli* O157:H7, *Listeria innocua*, *Saccharomyces cerevisiae*, *Aspergillus niger*, and *Staphylococcus aureus* [25]. Carvacrol microencapsulated in hydroxypropyl-β-cyclodextrin inhibited *E.coli* and *Salmonella* spp. at lower concentrations than non-encapsulated carvacrol [26]. These previous results, along with those presented herein, suggest that encapsulation could enhance antimicrobial action and decrease the minimal inhibitory concentration of carvacrol, which indicates that carvacrol encapsulation complexes could be useful as antimicrobial delivery systems.

### 3.2. Firmness and Weight Loss

**Figure 2:**
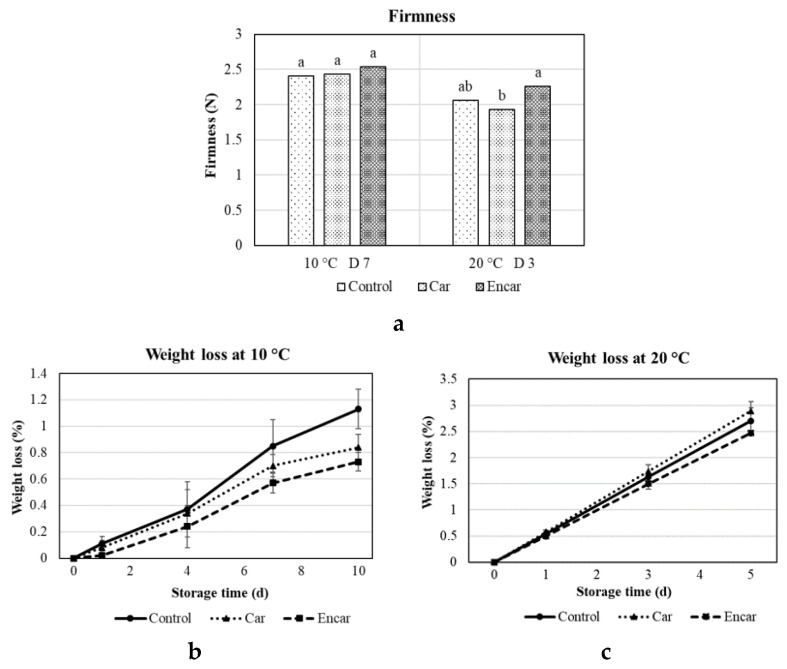
Effects of pectin-sodium alginate microencapsulated carvacrol (Encar) and non-encapsulated carvacrol (Car) on firmness (**a**) and weight loss (**b**,**c**) of fresh blueberries at 10 °C for up to 10 days, and 20 °C for up to 5 days. Bars with different letters within a storage temperature indicate significant differences using the Student’s t test (*p* < 0.05, *n* = 20).

The encapsulated carvacrol treated berries retained higher firmness and had lower weight loss (Figure 2). On average, the firmness of the encapsulated carvacrol treated berries was 17.3%, and 9.8% higher than non-encapsulated carvacrol treated fruit and control, respectively, after 3 days of storage at 20 °C (Figure 2a). Firmness is one of the most essential quality features for blueberries [27] and is determined by a number of factors governing cellular structure, including the mechanical and physiological properties of cells, fruit anatomy and cellular construction, biochemical changes in the cell wall, membrane integrity and turgor pressure [28].

A reduction in postharvest softening due to carvacrol treatments had been reported in other fruits, including cherry tomatoes [29], mandarin oranges [30], and blueberries [2]. In another study, carvacrol treated fruit had reduced enzyme activity of polyphenol oxidase and peroxidase, significantly contributing to the softening process [31], or reduced respiration rate and ethylene biosynthesis, reducing postharvest decay [2]. Generally, during fruit ripening, the changes in cell wall polysaccharides could change the chemical structure of pectin and decrease fruit firmness [32]. Since carvacrol inhibits the synthesis of cell wall proteins, it could conceivably appreciably delay fruit softening, and as a secondary effect, hinder cell wall penetration by pathogens and reduce postharvest decay [25].

The weight loss of blueberries treated with either encapsulated or non-encapsulated carvacrol were 0.73% and 0.84%, respectively, after 10 days of storage at 10 °C, and 2.47% and 2.89%, respectively, after 5 days of storage at 20 °C (Figure 2b,c). The control fruit stored at 10 °C and 20 °C displayed weight loss of 1.13% and 2.70% at the end of the weighed storage period (Figure 2b,c). The weight loss of fruit treated with encapsulated carvacrol was significantly lower than control after 7 days and 10 days of storage at 10 °C, and significantly lower than non-encapsulated carvacrol treated fruit after 5 days of storage at 20 °C (Figure 2b,c). A linear relationship between firmness and weight loss was established in blueberries [33]. Carvacrol inhibited microbial (bacteria and mold) growth thus maintained the integrity of cuticular wax [34], and prevented fruit from water evaporation or weight loss, which further maintained fruit turbulence and firmness. It was also proposed that a controlled-release carvacrol could reduce fruit metabolism, in addition to inhibiting weight loss and maintaining firmness [30]. Cherry tomatoes coated with the combinations of chitosan and carvacrol exhibited weight loss between 11.1% and 14.2% at the end of storage, and higher carvacrol concentrations corresponded to lower weight loss [29]. Carvacrol complexed in β-cyclodextrins and poly(lactic acid) maintained the weight of blackberries and raspberries up to 21 days [35]. The result of the current research suggests a similar consequence of carvacrol treatment causing a retention of fruit firmness and reduction in weight loss.

### 3.3. Sensory Evaluation

**Figure 3:**
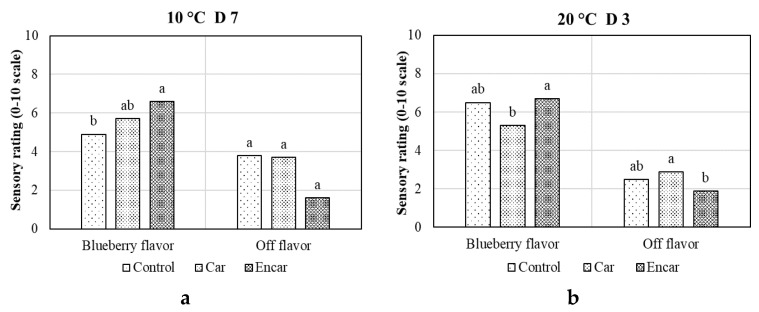
Effects of pectin-sodium alginate microencapsulated carvacrol (Encar) and non-encapsulated carvacrol (Car) on sensory properties of fresh blueberries after 7 days storage at 10 °C (**a**), and 3 days storage at 20 °C (**b**). Bars with different letters indicate significant differences using the Student’s t test (*p* < 0.05, *n* = 10).

Berries from the encapsulated carvacrol treatment showed the highest blueberry flavor and lowest off-flavor (Figure 3). The blueberry flavor from the encapsulated carvacrol treatment was significantly higher than control and non-encapsulated carvacrol treatment after 7 days storage at 10 °C, and 3 days storage at 20 °C, respectively (Figure 3). The encapsulated carvacrol treated berries showed significant lower off-flavor than non-encapsulated carvacrol treated fruit after 3 days storage at 20 °C (Figure 3b).

Higher off-flavor scores in the control fruit were probably associated with high microbial populations and, in the carvacrol direct application treated fruit, the off-flavor was most likely due to the high residue of carvacrol in the headspace of the package and on the fruit surface, which will be discussed later. The higher scores of blueberry flavor and low scores of off-flavor in microencapsulated carvacrol treated blueberries most likely resulted from the slow release of carvacrol over time, inhibiting microbial growth, reducing water loss and maintaining turgor, and increasing fruit firmness, without excessive accumulation of carvacrol on fruit surface. Our earlier experiment with blueberry partially supported this supposition [27]. Previous research with blueberry and other fruit also exhibited an improvement in sensory quality after carvacrol treatment, such as carvacrol significantly increasing the concentration of chlorogenic acid, and the major phenolic compound in fresh blueberries [3]. Kumquat treated with a chitosan coating incorporating carvacrol retained good sensory acceptability with higher nutritional components [36]; and a β-cyclodextrin-carvacrol inclusion complex was highly appreciated in sensory analyses in fresh mandarins [30]. Those improvements of sensory quality often associated with increased SSC and decreased TA [3,30]. However, in the current experiment, SSC (10.5–11.6%) and TA (0.58–0.72%) values were not significantly different among the treatments, indicating that they were not major contributors to the sensory evaluation results.

### 3.4. Carvacrol Residue and Volatile Profile

**Figure 4:**
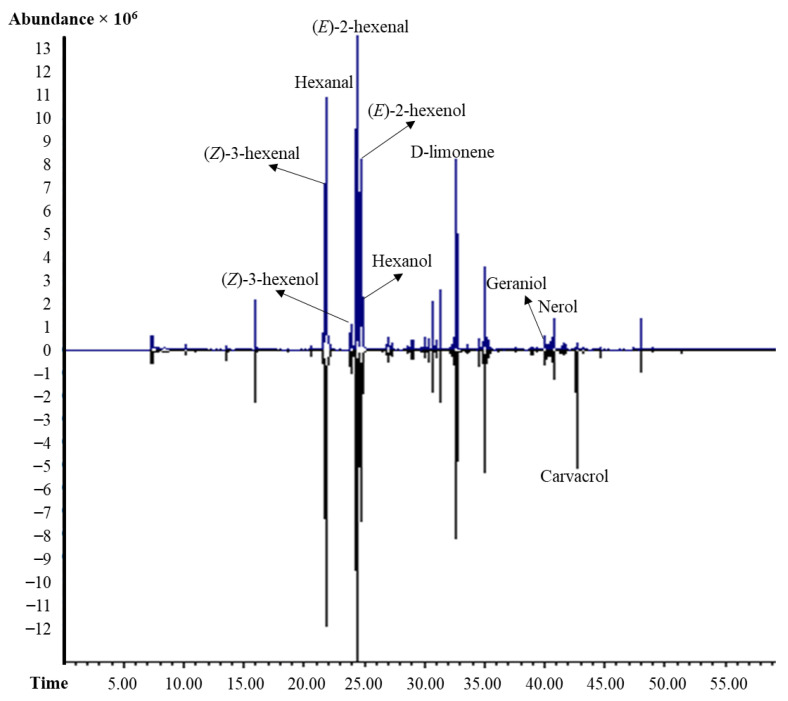
GCMS chromatograms of microencapsulated carvacrol (top) and non-encapsulated carvacrol (bottom) treated blueberries after 7 days storage at 10 °C. Control blueberries had similar chromatograms with no carvacrol detected.

The abundant volatile compounds in blueberries were (*Z*)-3-hexenal, hexanal, (*E*)-2-hexenal, (*E*)-2-hexenol, (*Z*)-3-hexenol, hexanol, D-limonene, geraniol, and nerol (Figure 4), similar to a previous report [37]. Residual carvacrol in the treated blueberries was 227.9 mg kg^−1^ after 7 days of storage at 10 °C in the non-encapsulated carvacrol treatment, and 5.17 mg kg^−1^ in the microencapsulated carvacrol treatment (Figure 4). Carvacrol flavor has been described as a spicy, warm odor, and ‘thymol-like’ [38], and the average odor detection threshold is 30.97 mg kg^−1^ in sunflower oil [39]. Based on this published data, and considering carvacrol in our study was in a hydrophobic medium, the estimated odor activity value (OAV, volatile concentration/the odor detection threshold) in non-encapsulated carvacrol treatment was higher than 7, explaining the off-flavor perceived by panelists (Figure 3), and described as a spicy or medicinal off-flavor. The OAV in encapsulated carvacrol treatment was less than 1, which explains the lack of off-flavor in these fruit.

Carvacrol treatments did not significantly affect the volatile profile of blueberries regardless of delivery method (Figure 4). Carvacrol metabolism occurs following two types of pathways: conjugation of the phenolic group with glucuronic acid and sulphate, and extensive oxidation of its methyl groups [5]. In this research, the fruit volatile profile was not affected by different carvacrol treatments regardless of delivery method and the substantial differences in the carvacrol residue, indicating carvacrol was not metabolized by the berries into other volatile compounds (Figure 4).

Proper carvacrol concentration in the clamshells and residue on the fruit surface are keys to success in finding a balance between enough to prevent microbial growth but not too much causing a persistent off-odor. Marin et al. [17] showed that when controlled-release thymol dosages were high enough (8 g), fungal decay in the packaged strawberries was reduced. However, low dose thymol (0.2 or 0.4 g) did not control decay [17]. Our preliminary experiments showed that in the commercial clamshells with a 2.83% opening ratio, controlled-release chlorine dioxide and essential oils never built up to a measurable level due to the over-venting, and this is why we have continually used an experimental clamshell with a 0.44% opening ratio in our controlled-release studies [17,27]. Bai et al. [15] analyzed the difference of buildup of water vapor in the two different clamshells, and revealed that the experimental clamshells prevented the ventilation cycle caused by the massive exchange of headspace gas in the commercial clamshells, and a similar mechanism was expected for the carvacrol in this experiment.

## 4. Conclusions

Controlled-release microencapsulated carvacrol improved blueberry fruit safety and quality. Encapsulated carvacrol treated berries retained higher firmness, had lower weight loss, and better flavor. These results indicate that pectin/sodium alginate controlled-release microencapsulated carvacrol could be used for the preservation of blueberries and other small fruit without causing any off-flavor, provided it is used in clamshells with lower vent opening ratio than commercial clamshells.

## Figures and Tables

**Table 1:** Effects of pectin-sodium alginate microencapsulated carvacrol (Encar) and non-encapsulated carvacrol (Car) on microbial populations of blueberries at 10 °C for up to 10 days, and 20 °C for up to 5 days, expressed in log CFU g^−1^.

Treatment	10 °C											
	Day 0			Day 4			Day 7			Day 10		
	ECP	BP	Y/MP	ECP	BP	Y/MP	ECP	BP	Y/MP	ECP	BP	Y/MP
Control	3.96	4.09	4.12	4.79 a	4.25 a	4.54 a	3.99 a	4.31	4.62 a	4.37	4.42 a	4.44 a
Car	3.96	4.09	4.12	4.32 b	3.89 b	3.88 b	0 b	4.11	3.85 b	4.16	4.01 b	3.04 b
Encar	3.96	4.09	4.12	4.25 b	3.78 b	3.82 b	0 b	4.19	3.19 c	4.34	3.86 c	2.37 c
**Treatment**	**20 °C**											
	**Day 0**			**Day 1**			**Day 3**			**Day 5**		
	ECP	BP	Y/MP	ECP	BP	Y/MP	ECP	BP	Y/MP	ECP	BP	Y/MP
Control	3.96	4.09	4.12	3.34	3.55 a	3.93 a	4.04 a	4.19 a	4.82 a	4.28 a	4.11 a	4.29 a
Car	3.96	4.09	4.12	3.19	3.81 a	3.82 a	0 b	3.59 b	3.77 b	3.85 b	3.67 b	3.71 b
Encar	3.96	4.09	4.12	3.11	2.27 b	3.01 b	0 b	3.61 b	3.57 b	3.79 b	3.76 b	3.89 b

Means followed by a different letter in the same column within a storage temperature were not significantly different using the Student’s *t* test (*p* < 0.05, *n* = 3). ECP: population of *E. coli*; BP: population of mesophilic aerobic bacteria; Y/MP: population of yeasts and molds.
